# A structural view of the SARS-CoV-2 virus and its assembly

**DOI:** 10.1016/j.coviro.2021.11.011

**Published:** 2022-02

**Authors:** Nathan J Hardenbrook, Peijun Zhang

**Affiliations:** 1 Division of Structural Biology, Wellcome Trust Centre for Human Genetics, University of Oxford, Oxford, OX3 7BN, UK; 2 Electron Bio-Imaging Centre, Diamond Light Source, Harwell Science and Innovation Campus, Didcot, OX11 0DE, UK; 3 Chinese Academy of Medical Sciences Oxford Institute, University of Oxford, Oxford, OX3 7BN, UK

## Abstract

The SARS-CoV-2 pandemic that struck in 2019 has left the world crippled with hundreds of millions of cases and millions of people dead. During this time, we have seen unprecedented support and collaboration amongst scientists to respond to this deadly disease. Advances in the field of structural biology, in particular cryoEM and cryo-electron tomography, have allowed unprecedented structural analysis of SARS-CoV-2. Here, we review the structural work on the SARS-CoV-2 virus and viral components, as well as its cellular assembly process, highlighting some important structural findings that have made significant impact on the protection from and treatment of emerging viral infections.

## Highlights

• Structural biology plays a vital role in SARS-CoV-2 vaccine and treatment.
• High-resolution structures of SARS-CoV-2 proteins and complexes have been obtained.
• *In situ* structures of SARS-CoV-2 virus and its assembly are visualized by cryoET.

**Current Opinion in Virology** 2022, **52**:123–134

This review comes from a themed issue on **Virus structure and expression**

Edited by **José R Castón** and **Adam Zlotnic**

For complete overview about the section, refer Virus structure & Expression (2022)

Available online 4th December 2021

**https://doi.org/10.1016/j.coviro.2021.11.011**

1879-6257/© 2021 The Authors. Published by Elsevier B.V. This is an open access article under the CC BY license (http://creativecommons.org/licenses/by/4.0/).

## Introduction

In November 2002, an outbreak of an atypical pneumonia struck the Guangdong province of China [1]. A novel coronavirus, later named SARS-CoV, was identified as the cause of the epidemic, with a case fatality rate of 9.7% [2, 3, 4, 5]. This was followed by the Middle eastern respiratory syndrome coronavirus (MERS-CoV) outbreak in 2012, with a very high case fatality rate of 34% [5]. In 2019, the world was hit by another strain of coronavirus, SARS-CoV-2. SARS-CoV-2 has a much lower fatality rate that increases steeply with age. However, it has a far higher transmission rate than SARS-CoV or MERS-CoV [5]. SARS-CoV-2 pandemic that struck in 2019 has left the world traumatized. With over 200 million cases, and 4 million deaths as of October 1st, 2021, this pandemic has made it abundantly clear how fast a deadly virus can overwhelm our current medical resources, and heavily impact the global economy and everyone’s life.

During this time, we have seen unprecedented collaboration and support amongst scientists to develop methods of testing, treatment, and produce effective vaccines against SARS-CoV-2 showing that vaccines can be created quickly in response to such a global crisis [6]. Structural knowledge of the virus and its viral components is critical for the development of novel treatments and vaccines. Structural biology has provided structural information for the development of vaccines for SARS-CoV-2 that utilize the spike, as well as the development of potential therapeutics targeting the main protease (M^Pro^) [7^••^,8^•^,^9^]. Other protein factors, such as the papain protease (PL^Pro^) and RNA-dependent RNA polymerase (RdRp) also present promising targets for therapeutic treatment [10, 11, 12,13^••^]. Following the outbreak, an astounding number of protein structures from the SARS-CoV-2 virus have been determined, among which over 1400 atomic models deposited in the RCSB protein databank (PDB) and 600 electron densities in the Electron Microscopy Databank (EMDB) (as of October 1st, 2021). These structures reveal how the virus infects its host and provide the basis for development of the COVID-19 vaccines and novel therapeutics [14,15,16^••^]. Here we review major structural efforts on the virus, viral components, and its assembly process.

## Molecular architecture of the SARS-CoV-2 virus

**Figure 1:**
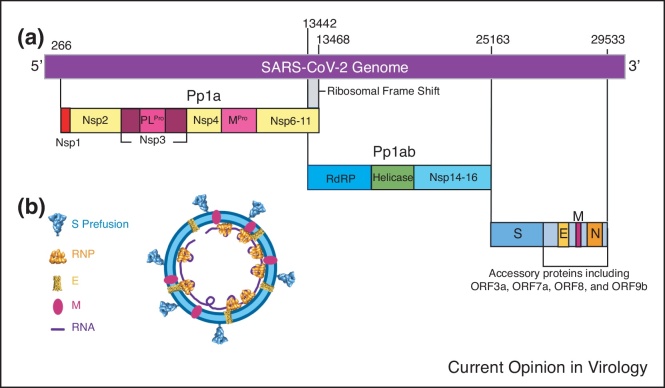
Organization of SARS-CoV-2. **(a)** Overall genomic organization of SARS-CoV-2. Non-structural proteins Nsp1-16 are expressed as polyproteins Pp1a and Pp1ab and are cleaved by internal M^pro^ and PL^Pro^ proteases. Structural proteins S, E, M, and N are encoded by their respective genes, interspaced with accessory proteins which include ORF3a. **(b)** A schematic model of SARS-CoV-2 virion with structural proteins indicated.

**Figure 2:**
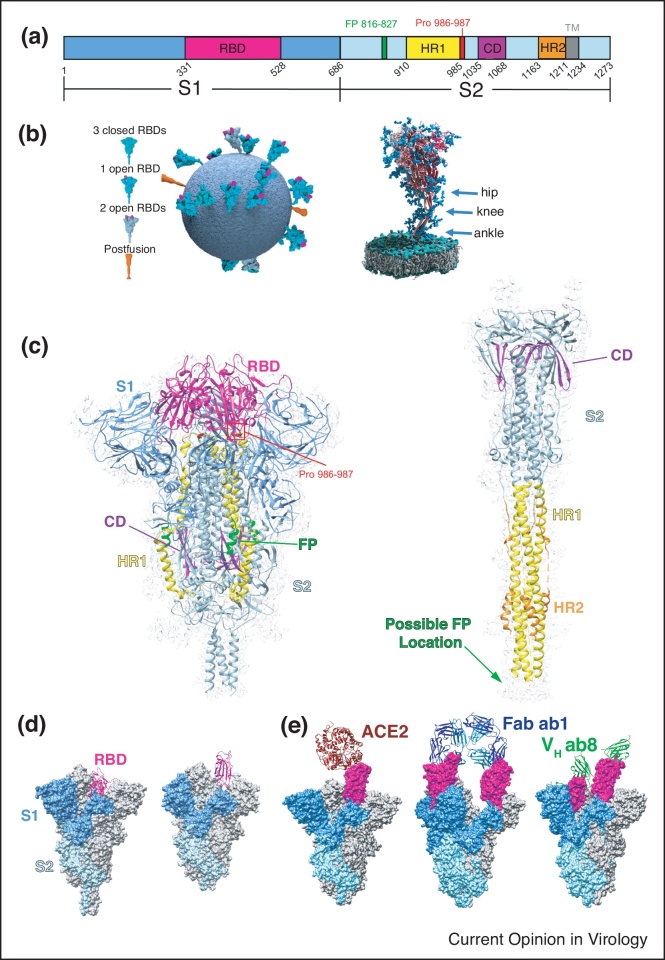
Structures of SARS-CoV-2 spikes. **(a)** Schematic representation of the SARS-CoV-2 S domains showing S1 subunit(blue), S2 subunit (light blue), Receptor binding domain (RBD, magenta), fusion peptide (FP, green), heptad repeat 1 (HR1, yellow), connector domain (CD, purple), heptad repeat 2 (HR2, orange) and the transmembrane region (TM, grey). **(b)** Model of the SARS-CoV-2 virion showing the conformations and flexibility of S on the surface of virions. Reprinted by permission from Springer Nature Customer Service Centre GmbH: Springer Nature, Nature, Ke *et al.* [23^••^]: Structures and distributions of SARS-CoV-2 spike proteins on intact virions. Copyright 2020 [19]. Three flexible hinges are marked by arrows on the right. Adapted from Ref. [26^•^] under CC BY 4.0. **(c)** Single-particle cryoEM structure of the S trimer in its prefusion (PDB 6XR8 and EMD-22292) and postfusion (PDB 6XRA and EMD-22293) conformations with structural components colored to match the color scheme in (a) [32^••^]. **(d)** Comparison of S with RBD in the ‘down’ (left, PDB 6XR8) [32^••^] and ‘up’ (middle, 6VYB) [62] conformations. **(e)** CryoEM structures of S bound to ACE2 (left, PDB 7DF4) [34^•^] with RBD in the ‘up’ conformation, S_N501Y_ mutant bound by Fab ab1 (middle, PDB 7MJJ) [33] with RBD in the ‘up’ conformation, and S_N501Y_ bound by V_H_ ab8 in both the RBD ‘up’ and ‘down’ conformations (7MJH) [33].

Recent breakthroughs in cryo-electron tomography (cryoET) and subtomogram averaging (STA) have allowed for unprecedented structural analysis of molecular complexes in their native state to near-atomic resolution [17, 18, 19, 20, 21, 22]. Several groups have used cryoET STA to image intact SARS-CoV-2 virions, providing insight into their molecular architecture and organization [23^••^,24^••^,25^•^,26^•^]. SARS-CoV-2 virions take roughly spherical or ellipsoidal shape with an average diameter of 108 ± 8 nm [24^••^,25^•^]. The external surface of the virion is covered with surface spike proteins (S) in a primarily prefusion conformation, with a small fraction of S in its postfusion form (Figure 1, Figure 2b) [23^••^,24^••^,25^•^,26^•^]. S appears as a flexible head on a stalk, able to tilt up to 90° relative to the membrane, with the majority appearing to tilt less than 50° (Figure 2b) [23^••^,24^••^,26^•^]. This flexibility is provided by hinges in the stalk region (Figure 2b) [24^••^,26^•^]. The surface of the S trimer is heavily glycosylated, with each S monomer containing 22 glycosylated sites [27]. This glycan coat, paired with the flexibility of SARS-CoV-2 spikes, enables them to scan the host cell surface and bind with the cell receptor ACE2 while shielded from neutralizing antibodies [24^••^,26^•^]. The viral outer membrane contains the membrane protein (M) and envelope protein (E) (Figure 1b). Within the lumen of the virion are the ribonucleoprotein (RNP) complexes consisting of the nucleocapsid protein (N) and the viral genome, responsible for the packaging of the RNA genome of the virus, with estimates of 30-35 RNPs per virion (Figure 1b) [24^••^]. The non-structural proteins (Nsps) Nsp1-16 are produced from self-cleavage of the precursor polyproteins Pp1a and Pp1ab by viral proteases [11]. PL^Pro^ (Nsp3) cleaves three sites resulting in free Nsp1-3, while M^Pro^ (Nsp5) is responsible for the remaining 11 cleavage sites [11]. This allows the Nsps to perform their functions in the host cell ranging from RdRp (Nsp12) and helicase (Nsp13) functions, generating double membrane vesicles for viral genome replication, transcription, and RNA transport (Figure 1a).

## Spike glycoprotein

The coronavirus surface S glycoprotein is a ∼600 kDa trimer, one of the largest known class 1 fusion proteins. Located on the outer envelope of the virion, it plays a critical role in viral infection through recognition of the host cell receptors and by mediating the fusion of the viral and host cell membranes. S also has been shown to elicit a strong immune response, making it the primary target for the recently developed vaccines for SARS-CoV-2 necessary to stem the COVID-19 pandemic [28, 29, 30, 31]. The SARS-CoV-2 S gene encodes a ∼1300 amino acid precursor protein which is then activated through proteolytic cleavage into an amino (N)-terminal S1 subunit (∼700 amino acids), and a carboxyl (C)-terminal S2 subunit (∼600 amino acids) with a single transmembrane (TM) region anchor (Figure 2a). The S1 and S2 subunits form a heterodimer, that in turn oligomerize into a trimer resulting in the formation of the surface spike on the virion (Figure 2b–c) [32^••^].

The S1 subunit consists primarily of an N-terminal domain (NTD) and a receptor binding domain (RBD), as well as two C-terminal domains (Figure 2a,c). S2 consists of a fusion peptide, heptad repeat (HR1), central helix region, connector domain (CD), heptad repeat 2 (HR2), and the transmembrane region (Figure 2a,c) [32^••^]. In its prefusion form, the S protein appears to have two conformations: the ‘RBD down’ conformation and the ‘RBD up’ conformation (Figure 2d). In its trimeric prefusion form, the ‘RBD’ down, ‘one RBD up’, and ‘two RBD up’ conformations have been observed [23^••^,24^••^,32^••^]. During infection, the RBD of SARS-CoV-2 binds to angiotensin converting enzyme 2 (ACE2) on the surface of target cells before undergoing viral uptake and fusion (Figure 2e) [33,34^•^,^35^, ^36^, ^37^]. ACE2 has only been shown to bind RBDs in the ‘up’ conformation [33,34^•^]. The RBD site has been shown to be of importance in neutralizing SARS-CoV-2 by targeting S with neutralizing antibodies [33,38,39^•^,40^•^,^41^, ^42^, ^43^, ^44^, ^45^, ^46^, ^47^, ^48^, ^49^, ^50^, ^51^]. The cryoEM structures of most antibodies bound to S have least one RBD in the ‘up’ conformation, similar to ACE2 (Figure 2e) [33,38,39^•^,^52^,^53^]. However, several other antibodies and antibody fragments such as V_H_ ab8 can bind RBDs in the ‘down’ conformation (Figure 2e) [33,41,45,54]. Other antibodies bind to other regions of S, such as the NTD [55, 56, 57, 58]. While the glycosylation of S is thought to shield it from antibody recognition, some neutralizing antibodies can still bind to glycan-containing epitopes, allowing immune response [59, 60, 61]. Previous studies of SARS-CoV have found two proline substitutions at residues 986 and 987 (Figure 2a,c) stabilize S in its prefusion form, which elicits a strong immune response [7^••^,^62^]. S stabilized with this two-proline mutation has been used in the development of both the Moderna and Pfizer mRNA vaccines [14,15].

During infection, the S1 subunit is shed and S2 undergoes a large conformational change compared to its prefusion state (Figure 2c) [32^••^,^63^]. This structural re-arrangement brings the fusion peptide and transmembrane domain together at the same end of the spike molecule, resulting in the insertion of the fusion peptide into the host membrane (Figure 2c) [32^••^,^63^]. HR1 and CD form an extended, three-stranded coiled-coil (Figure 2c) [32^••^]. During this transition, it is thought that the glycans mask the accessible surface of the S2 subunit, protecting it from antibody recognition [27]. For a more detailed review of the SARS-CoV-2 spike, please refer to Zhang, J., *et al.* (2021). ‘Structure of SARS-CoV-2 spike protein.’ *Current Opinion in Virology* **50**: 173–182.

## Envelope protein

Along with M, the coronavirus E protein is one of the major membrane components in SARS-CoV-2. E is a small, 8.5 kDa protein consisting of 75 amino acid residues. In coronaviruses, E is a cationic selective viroporin, forming a channel across the endoplasmic reticulum-Golgi intermediate compartment (ERGIC) membrane. In SARS-CoV, E mediates the budding and release of viruses [64^•^]. Deletions of E have been shown to attenuate the virus, while mutations abolishing channel activity reduce pathogenicity [65]. This provides a target for potential antiviral drug development as well as a potential vaccine candidate in SARS-CoV-2.

**Figure 3:**
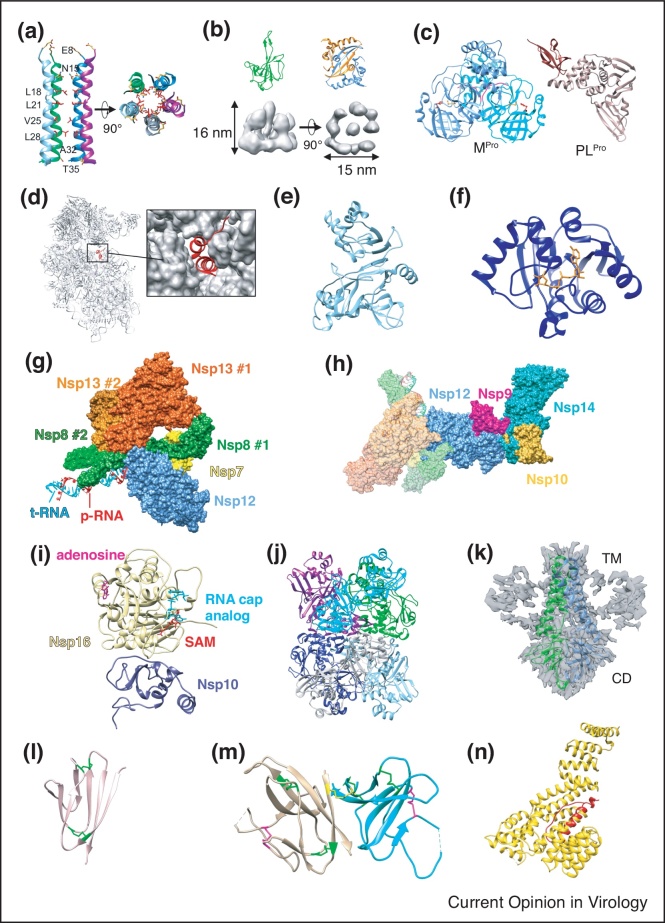
Structures of other viral components. **(a)** Top and side view of the transmembrane domain of SARS-CoV-2 E in lipid bilayers with hydrophobic residues in red (PDB 7K3G) [64^•^]. **(b)** Crystal structures of N NTD (green, PDB 7CDZ) [67] and CTD dimer (blue and orange, PDB 7CE0) [67] and 13.1 Å cryoET/STA density map of the RNP complex (grey, EMD-30429) [24^••^]. **(c)** Crystal structure of the SARS-CoV-2 Main Protease dimer (M^pro^, left, PDB 6Y2E) [8^•^] and Papain-like Protease (PL^pro^, right, PDB 6WZU) [11]. The M^pro^ catalytic dyad His41 and Cys145 are shown in red and orange, respectively and the N-terminal finger of each protomer is shown in magenta. The PL^pro^ ubiquitin-like domain is shown in brown and catalytic domain in rose. **(d)** CryoEM structure of Nsp1 bound within the mRNA entrance channel of 40S ribosome subunit, with a close-up surface representation of the ribosome showing Nsp1 helices within entrance channel (PDB 6ZOK) [77]. **(e)** Crystal structure of Nsp2_1-276_ (light blue) (PDB 7EXM) [80]. **(f)** CryoEM structure of the Nsp3 macrodomain (blue) in complex with ADP-ribose (orange) (PDB 6W02) [82]. **(g)** CryoEM structure of the SARS-CoV-2 Nsp13-RTC complex with RNA (PDB 6XEZ) [88]. **(h)** CryoEM structure of the cap(0)-RTC complex consisting of Nsp9/10/14 bound to the Nsp13-RTC complex (PDB 7EIZ) [95^••^]. **(i)** Crystal structure of the Nsp16 (light yellow) and Nsp10 (dark blue) heterodimer with an RNA cap analog (cyan) and *S*-adenosyl methionine or SAM (red) with an occupied adenosine binding pocket (magenta) (PDB 6WKS) [98]. **(j)** Crystal structure of the Nsp15 hexameric endonuclease (PDB 6VWW) [99]. **(k)** CryoEM structure of ORF3a dimer in lipid nanodiscs. TM: Transmembrane domain. CD: Cytosolic Domain (PDB 6XDC and EMD-22136) [102]. **(l)** Crystal structure of ORF7a (pink) (PDB 7CI3) [103]. Disulfide bonds shown in green **(m)** Crystal structure of the ORF8 homodimer (tan and cyan) (PDB 7JTL) [104]. Disulfides conserved with ORF7a (green), intermolecular disulfide (yellow) and ORF8-specific disulfide (magenta) are shown. **(n)** CryoEM structure of ORF9b (red) bound to TOM70 (gold) (PDB 7KDT) [106].

Despite its importance, until recently the E protein structure remained elusive. An NMR structure of the transmembrane domain structure of SARS-COV-2 E was determined using solid state NMR in phospholipid bilayers [64^•^]. This work reveals that E consists of a compact and rigid homopentameric helical bundle transmembrane domain (Figure 3a). The central portion of the TM domain contains four hydrophobic residues lining the core, narrowing the radius to ∼2 -Å. As this would only permit a single file of water molecules and partially dehydrate any ions that move through the pore, this structure may represent a closed state of SARS-CoV-2 E [64^•^]. The E transmembrane domain provides a potential novel target for small molecules which could target the polar Asn15 or the acidic Glu8 residues to occlude the N-terminal entrance of the channel (Figure 3a) [64^•^].

## Nucleocapsid protein

The major protein component of the SARS-CoV-2 inside of the virion is the nucleocapsid (N) protein. N is responsible for binding the genomic RNA within the virion and packaging it into the ribonucleoprotein (RNP) complex. N proteins have a variety of functions beyond packaging, with the SARS-CoV-2 N protein found to interfere with RNA interference (RNAi) [66] and function as a viral suppressor of RNAi (VSR) in cells (Table 1).

**Table 1:** SARS-CoV-2 gene products and structures (PDB and EMDB)

Protein or complex	PDB ID	EMD ID	Ref.
Nsp1	6ZOK	EMD-11321	[77]
Nsp2_1-276_	7EXM		[80]
Nsp3 Macro Domain	6W02		[82]
Nsp3 PL^Pro^	6WZU		[11]
Nsp5 M^Pro^	6Y2E		[8^•^]
Nsp13-RTC	6XEZ	EMD-22160	[88]
cap(0)-RTC	7EIZ	EMD-31146	[95^••^]
Nsp10/Nsp16	6WKS		[98]
Nsp15 NendoU	6VWW		[99]
S Prefusion (RBD Down)	6XR8	EMD-22292	[32^••^]
S (Postfusion)	6XRA	EMD-22293	[32^••^]
S Prefusion(one RBD up)	6VYB	EMD-21457	[62]
S Prefusion-ACE2	7DF4	EMD-30661	[34^•^]
S_N501Y_ Prefusion-Fab ab1	7MJJ	EMD-23875	[33]
S_N501Y_ Prefusion-V_H_ ab8	7MJH	EMD-23873	[33]
E	7K3G		[64^•^]
N NTD	7CDZ		[67]
N CTD	7CE0		[67]
N (cryo-STA)		EMD-30429	[24^••^]
Orf3a	6XDC	EMD-22136	[102]
Orf7a	7CI3		[103]
Orf8	7JTL		[104]
Orf9b	7KDT	EMD-22829	[106]

In SARS-CoV-2, the N protein consists of the three intrinsically disordered regions: the N-arm, central linker region (LKR), and C-tail, as well as the two structural domains: the NTD (Figure 3b, green) and CTD (Figure 3b, orange/blue) [67]. Previous work on SARS-CoV has shown that the NTD serves as the RNA-binding domain, while the CTD functions as a dimerization domain [68].

In SARS-CoV-2, N protein forms dimers through the CTD interactions (Figure 3b) [67,69, 70, 71]. N proteins form RNP complexes with the viral genome. These RNPs are thought to be linked to neighboring in a ‘beads on a string’ manner [72]. CryoET STA has revealed a reverse G-shaped architecture of RNPs in intact virions (Figure 3b, grey) [24^••^]. Because of the limited resolution, it remains unclear how the individual N proteins and RNA are organized within the vRNP [24^••^,^72^]. Fitting in previously determined structures of the NTD and CTD domains from SARS-CoV into the cryo-STA map seems to indicate a decamer of N proteins forming the base unit of the RNP, with the electrostatic potential on the decamer surface suggesting the RNA winds around the N protein dimers [24^••^], although further work and analysis remains necessary. This ‘beads on a string’ mechanism of genome packaging would maintain the high steric flexibility of the vRNPs necessary to allow the unusually large genome to be packaged efficiently within the budding virions [72].

## Main protease and papain-like protease

One attractive drug target for SARS-CoV-2 is non-structural protein 5 (Nsp5), the main protease (M^Pro^). M^Pro^, a 3C-like protease, is responsible for processing 11 M^Pro^-specific sites on the two SARS-CoV-2 polyproteins Pp1a and Pp1ab into 16 nonstructural proteins (Nsp1-16) [73,74]. The crystal structure of M^Pro^ was recently determined (Figure 3c, blue and cyan) [8^•^,16^••^]. M^Pro^ consists of three domains, a chymotrypsin-like domain I and piconavirus 3C protease-like domain II, as well as domain III, consisting of 5 antiparallel α-helices that regulates dimerization [8^•^]. Dimerization is essential for the enzymatic function of M^Pro^, with the N-terminal residue (N-finger) in each protomer squeezed between domains II and III of the parent protomer and domain II of the partner protomer (Figure 3c), interacting with Glu166 of the partner and shaping the substrate binding pocket [8^•^,^75^]. The binding pocket of M^Pro^ is located between domains I and II, with a Cys-His catalytic dyad (Figure 3c) functioning as the active site. M^Pro^ targets a Leu-Gln↓(Ser, Ala, Gly) recognition sequence (with ↓ denoting the cleavage site) [8^•^]. As no known human proteases have the same specificity as M^Pro^, M^Pro^ appears to be an ideal target for therapeutic development [8^•^,16^••^,^73^]. The structure of M^Pro^ was then used to develop an α-ketoamide inhibitor that targets the active site of SARS-CoV-2 M^Pro^ as the basis of therapeutic development [8^•^]. In addition, the structure of M^Pro^ was determined with N3, a mechanism-based inhibitor and used for further inhibitor discovery using *in silico* analysis [16^••^].

A second, papain-like protease (PL^Pro^), encoded in Nsp3, is the protease responsible for cleaving the remaining three cleavage of polyproteins [10]. The crystal structure of PL^Pro^ has also been determined [11,76], showing that PL^Pro^ contains two domains: a small, N-terminal ubiquitin-like domain, and a catalytic domain with a ‘thumb-palm-fingers’ architecture (Figure 3c). The catalytic active site sits between the thumb and palm domains and contains a canonical cysteine protease catalytic triad, recognizing a Leu-X-Gly-Gly↓(XX) sequence [11]. PL^Pro^ can recognize the C-terminal sequence of ubiquitin. This makes development of a PL^Pro^ inhibitor more challenging, as care must be taken to ensure any inhibitor doesn’t interfere with host deubiquitinases [73].

## Other non-structural proteins

The structures of several other non-structural proteins from SARS-CoV-2 have been reported as potential targets for therapeutics. Nsp1 functions as a host shutoff factor, binding the mRNA entrance channel of ribosome complexes. A cryoEM structure of Nsp1-ribosome shows its C-terminal forming two α-helices binding within the entrance channel of the 40S subunit (Figure 3d) [77, 78, 79]. Helix 1 (residues 153–160) interact with the ribosome helix 18 through hydrophobic interactions, while helix 2 (residues 166–178) interact with the phosphate backbone of helix 18 through conserved arginine residues R171 and R175, allowing it to inhibit translation of host mRNA [77, 78, 79].

While the full-length structure of Nsp2 remains undetermined, recently the N-terminal domain of Nsp2 (Nsp2_1-276_) has solved [80]. This structure reveals the Nsp2_1-276_ structure to be a novel zinc finger domain consisting of three zinc fingers. ZnF1, ZnF2, and ZnF3 (Figure 3e) [80]. A large, positively charged region on the surface of Nsp2_1-276_ was then shown to be able to bind to dsDNA. By chelating the Zn with EDTA and using mutagenesis, Nsp2_1-276_ appears to bind to DNA through this charged surface, with the zinc fingers not being directly involved [80]. While this provides insight into the potential function Nsp2_1-276,_ the role of Nsp2 in SARS-CoV-2 infection remains unknown [80].

Along with PL^Pro^, Nsp3 contains a macrodomain responsible for removal of ADP-ribose from ADP-ribosylation sites during infection, potentially playing an important role in disrupting host ADP-ribosylation [81, 82, 83]. As ADP-ribosylation has been linked to innate immune response, this macrodomain may provide an attractive target for drug development. This macrodomain has a baseball glove-like structure, with an ADP-ribose-binding pocket (Figure 3f) [81, 82, 83]. A structural comparison of the binding site crystalized with a variety of substrates suggests high structural plasticity within the binding site, presenting an opportunity for rational targeting of small molecule inhibitors [84]. This pocket has been shown to bind GS-441524, a remdesivir metabolite, supporting the hypothesis that the macrodomain represents a promising drug target [84].

Nsp12, the RdRp, is essential for the synthesis of viral RNA and the primary target for RNA analog therapeutics such as remdesivir [12,13^••^,^85^]. On its own, Nsp12 has low polymerase activity. Upon addition of Nsp7 and Nsp8 cofactors and formation of a holo-RdRp:RNA complex with scaffold RNA made up of template RNA (t-RNA) and primer RNA (p-RNA), the polymerase activity of Nsp12 is greatly improved [86]. The holo-RdRp:RNA complex consists of a single Nsp7/Nsp8 heterodimer bound to Nsp12, as well as a single Nsp8 at a separate Nsp12 binding site [13^••^,^85^, ^86^, ^87^]. CryoEM structures of the holo-RdRp complex with its cofactors Nsp7 and Nsp8 reveal that Nsp12 (Figure 3g blue) contains an RNA polymerase domain as well as a nidovirus RdRp-associated nucleotidyltransferase (NiRAN) domain connected by an interface domain. The Nsp7-Nsp8 #1 heterodimer (Figure 3g, yellow and dark green) binds above the thumb subdomain of Nsp12, sandwiching Nsp12 finger extension loops between them and stabilizing this conformation. This interaction is primarily mediated by Nsp7, with Nsp8 contributing very little to the interaction with the polymerase domain. Nsp8 #2 (Figure 3g, green) binds to the finger subdomain of the Nsp12 polymerase domain and interacts with the interface domain of Nsp12 [86]. Nsp8 contains helical N-terminal extensions which interact with the RNA as it exits the complex, potentially promoting polymerase activity by stabilizing the exiting RNA [13^••^].

Nsp13 is a helicase that interacts with the holo-RdRp:RNA complex, forming the Nsp13-replication-transcription complex (Nsp13-RTC) essential for replication and transcription (Figure 3g) [88, 89, 90, 91]. Nsp13 contains two canonical RecA ATPase domains, as well as three domains unique to nidovirus helicases: an N-terminal zinc-binding domain (ZBD), a stalk, and a 1b domain. The Nsp13-RTC complex can exist in two isoforms, with either a single Nsp13 or two Nsp13 proteins bound (Figure 3g) [88, 89, 90, 91]. The Nsp13 ZBD domains interact with the Nsp8 N-terminal extensions. Nsp13 #1 (Figure 3g, dark orange) also interacts with the thumb domain of Nsp12 (Figure 3g, blue), while the first RecA domain interacts with the head of Nsp8 #1 (Figure 3g, dark green) as well as Nsp7 (Figure 3g, yellow). The overall architecture of the Nsp13-RTC complex places the Nsp13 RNA-binding channel directly in the path of t-RNA strand. The holo-RdRp complex translocates in the 3′-5′ direction, while the Nsp13 helicase is positioned to translocate on the RNA strand in the 5′-3′ direction, opposite the RdRp. This is thought to provide backtracking along the t-RNA and play a role in maintaining transcription-replication fidelity [88,91].

The Nsp9 in its crystal structure forms a dimer, with each monomer containing a unique fold limited to coronaviruses [92]. The structure consists of an enclosed six-stranded β-barrel with outward projecting loops connecting the β-strands with a projected N-terminal β-strand and a C-terminal α-helix make up dimerization interface, allowing it to dimerize. Nsp10 from SARS-CoV-2 is a non-classic zinc finger protein, containing two zinc finger motifs. Nsp10 acts as a co-factor, necessary for stimulation of Nsp14 and Nsp16 [93]. Nsp14 is a bifunctional protein, consisting of an N-terminal exoribouclease domain (ExoN) and a C terminal domain guanine-NT-MTase involved in caping [94,95^••^]. The overall structure of Nsp10/Nsp14-ExoN consists of the Nsp14-ExoN leaning along the Nsp10 monomer, with peripheral regions of Nsp14-ExoN interacting with most regions of Nsp10 [94]. Nsp10/14 associate with the Nsp13-RTC complex, mediated by an Nsp9-Nsp12 interaction, forming a cap(0)-RTC complex (Figure 3h) [90,95^••^]. This cap(0)-RTC complex can form dimers, positioning the Nsp14 ExoN domain facing the Nsp12 reaction center, revealing a potential mechanism for Nsp14 to exert its proofreading activity [95^••^].

Nsp16 is *S*-adenosylmethionine-dependent methyltransferase (SAM-MTase) essential for methylation of the viral RNA cap [96,97]. The overall structure of Nsp10/Nsp16 is of an Nsp16 monomer on top of an Nsp10 monomer [96]. Nsp16 takes on a canonical SAM-MTase fold, containing an RNA-binding groove and a *S*-adenysylmethionine (SAM) binding pocket occupied by SAM. A crystal structure of Nsp10/Nsp16 complexed with an RNA cap analog in the RNA-binding groove revealed the organization of the catalytic pocket in the presence of SAM and substrate (Figure 3i). This structure also showed an adenosine binding pocket opposite the catalytic pocket, not found in any other strains of coronavirus [98].

The Nsp15 (NendoU) of SARS-CoV-2 forms a hexameric endonuclease with a uridine specificity [99,100]. Nsp15 contains three domains: an N-terminal oligomerization domain, a middle domain, and an endoU catalytic domain [99]. Crystal structures reveal that Nsp15 oligomerizes into a hexameric form, made up of a dimer of trimers with the endoU catalytic domains located on opposite ends of the hexamer (Figure 3j). While its role in SARS-CoV-2 infection remains unclear, studies in other viruses suggest it may have multiple cleavage targets important for accumulation of viral RNA and preventing RNA-activated immune responses [100]. The cryoEM structures of Nsp15 both in presence and absence of 5′-UMP reveal that in the absence of substrate, the endoU domain appears to wobble, resulting in a loss of local resolution. This series of cryoEM and crystal structures of Nsp15 provide insight into how it targets RNA and could provide a drug target for nucleotide analogs such as Tipracil [100,101].

## Open reading frame accessory proteins

ORF3a from SARS-CoV-2 is a conserved protein across the *Sarbecovirus* subgenus, which includes SARS-CoV. ORF3a has been implicated in apoptosis and inhibition of autophagy. ORF3a has been proposed form an ion channel, the second viroporin in the SARS-CoV-2 genome. However, the function of ORF3a during infection remains unknown. The cryoEM structure of ORF3a was recently determined in lipid nanodiscs, revealing that ORF3a forms a dimeric or tetrameric ion channel [102]. ORF3a is composed of a transmembrane domain (TM) of three helices per protomer, TM1, TM2, and TM3 that connect to a cytosolic domain (CD) extending into the cytosol (Figure 3k). In the dimeric form, two of these protomers come together to form the ORF3a ion channel. The CD is made up of eight-stranded β-sheet sandwich, with the inner sheets from each protomer forming a stabile hydrophobic core. In its tetrameric form, two ORF3a dimers come together through interactions between TM3/CD linker region, as well as β1/β2 of neighbouring dimers.

In ORF3a, the lower half of the TM region contains a polar cavity with a lower tunnel, open to the cytosol, and an upper tunnel, likely open to the membrane. While most ion channels contain a central pore, in the case of ORF3a the extracellular TM region forms a hydrophobic seal [102]. Some ion channels have evolved pathways of external groves or tunnels on membrane-facing surfaces of the channel. ORF3a contains a distinct membrane-facing hydrophilic groove between TM2 and TM3, connected to the upper tunnel. Mutations in this region alter ion permeability, supporting the hypothesis of these external grooves are involved in ion transport [102]. As deletions of ORF3a have lowered viral titer and mortality in mice, this may provide a target for novel therapeutic development.

Several other small open reading frame protein structures have also been recently determined.

The crystal structure of the ectodomain of ORF7a has recently been determined, revealing an Ig-like fold structure consisting of seven β-strands organized into two tightly packed β-sheets stabilized by two disulfide bonds (Figure 3l) [103]. ORF7a has been shown to interact with CD14^+^ monocytes with high efficiency [103]. While it functions as an immunomodulating factor and triggers an inflammatory response, the mechanism of ORF7a’s interaction with CD14^+^ remains unknown [103].

ORF8 has been shown to disrupt IFN-I signalling in cells, as well as downregulate MHC-I. The crystal structure of ORF8 has been determined, revealing a homodimeric Ig-like fold (Figure 3m) [104]. This dimer is linked by an intermolecular disulfide bond. This Ig-like fold is stabilized by two disulfide bonds conserved between ORF7a and ORF8. ORF8 also contain an ORF8-specific region distinct from other Ig-like folds, containing a third ORF8-specific disulfide bond [104]. While many interactors for ORF8 have been identified, its mechanism of action remains unclear, necessitating more structural work of ORF8 in complex with host factors [104].

The structures of ORF9a provide structural insight into its mechanism for interfering with type 1 interferon immune response by targeting TOM70 [105,106]. TOM70 forms a surface receptor for the translocase of the outer membrane (TOM) complex in mitochondria, playing a key role in relaying antiviral signalling from mitochondrial antiviral signalling (MAVS) to the TANK-binding kinase 1 through recruitment of protein binding heat shock protein 90 (Hsp90), ultimately resulting in interferon response [105]. Upon binding to TOM70, ORF9b takes on a helical conformation that binds within a deep pocket of TOM70 C-terminal domain (CTD) (Figure 3n) [105,106]. This binding appears to stabilize TOM70 and allosterically inhibit recruitment of Hsp90, ultimately suppressing interferon response [105].

## Virus assembly in the context of host cell

**Figure 4:**
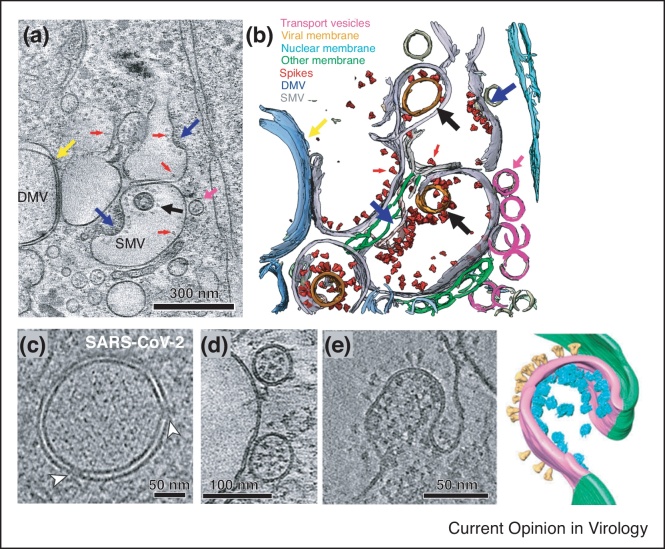
*In situ* cryoET of SARS-CoV-2 assembly process. **(a)**,**(b)** Tomographic slice of cryoFIB lamella depicting SARS-CoV-2 assembly and density segmentation, showing DMV portals (yellow arrow), assembling viruses (blue arrow), assembled virus (black arrow), viral spikes on SMV membranes (red arrows), and transporting vesicles around the assembly site (pink arrow). Adapted from Ref. [107^••^] under CC BY 4.0. **(c)** Tomographic slices revealed that pore complexes were present in fixed SARS-CoV-2–induced DMVs (white arrowheads) Reprinted from Ref. [108^•^] under CC BY 4.0. **(d)** Spike-filled transport vesicles in close proximity to a SMV Reprinted from Ref. [107^••^] under CC BY 4.0. **(e)** CryoET tomogram and 3D volume rendering of the early virion budding stage. S and vRNPs accumulate at the lumenal membrane. 3D volume rendering is shown with cellular and viral membranes in green and magenta, respectively, with S (yellow) and vRNP (cyan) represented as subtomogram averages Reprinted from Ref. [72] under CC BY 4.0.

One of the major advantages provided by advances in cryoET is cryo-focused ion beam scanning electron microscopy (cryoFIB/SEM). CryoFIB/SEM uses a focused ion beam to create 150−250 nm thick cell lamella, which can then be imaged using cryoET to determine macromolecular complex structures *in situ* [17]. This method was used for imaging the SARS-CoV-2 virions at different stages over the course of infection, allowing high resolution characterization of viral structure and replication (Figure 4a–b) [72,107^••^]. Using cryoFIB/SEM, double membrane vesicles (DMV) in fixed SARS-CoV-2 infected cells were revealed to contain multiple copies of a membrane-spanning pore complex (Figure 4c) thought to be composed of Nsp3 and other unknown proteins [107^••^,108^•^]. Newly synthesized RNA is hypothesized to be transported out of DMVs through the transmembrane portals for subsequently protein production and virus assembly [72,107^••^]. S is transported in its trimeric prefusion form to assembly sites via small transport vesicles that then fuse with single membrane vesicles (SMV) where virus assembly takes place (Figure 4d) [107^••^]. CryoET imaging of early budding events revealed a positively curved membrane decorated with S on the luminal side, and vRNPs on the cytosolic side (Figure 4e) [72]. S clusters with the SMV near the electron-dense areas with encapsidated RNPs, ultimately leading to budding [107^••^]. In SMVs, S trimers show a polarized distribution and is likely mobile, allowing them to redistribute during the budding process [72].

## Future perspective

The SARS-CoV-2 pandemic has brought the importance of scientific research to the forefront of the media and public’s view. The unprecedented collaboration has given us the tools and insights needed to develop not one, several vaccines in record time. The two-proline mutation that was identified in previous MERS-CoV and SARS-CoV work to stabilize S in its prefusion conformation [109] has been used in the development of both the Pfizer and Moderna mRNA vaccines, increasing the efficacy [7^••^,^14^,^15^]. CryoET has also been shown to be valuable in validating the post-translational processing and glycosylation of S in the ChAdOx1 nCoV-19 vaccine in human cells, providing a useful tool for checking future vaccine efficacy [110].

However, as the virus continues to spread and mutate, further research will be important in determining new vaccine and therapeutic targets, as it is unlikely that we will be able to eradicate SARS-CoV-2 anytime soon. The structure of M^Pro^ has already resulted in the direct design of inhibitors, as well as being used for virtual screening of thousands of compounds for potential inhibitors [8^•^,16^••^]. Pfizer has recently developed a novel M^Pro^ inhibitor, PF-07321332, the first orally administered M^Pro^ inhibitor to begin clinical trials [111]. Structures of S from variants could provide the basis for future vaccine development and provide insight into how variants evade immune response [112]. Structural work on the RdRp bound by nucleoside analogs presents it as a promising target for novel therapeutic development. Most recently, the inhibitor molnupiravir which causes an error catastrophe during replication, has been approved for use in the UK (as of 4th November 2021) [113].

Other proteins, such as Nsp3 macrodomain, also provide promising candidates for therapeutic development. As the Nsp3 macrodomain has been shown to bind remdesivir metabolite, it is a prime therapeutic target [84]. E also provides an interesting target, as deletions of E or blocking abolishing channel activity have shown promise in SARS-CoV [65]. Small molecules designed to target the acidic or polar residues at the N-terminal could provide an effective target [64^•^]. Structural work on PL^Pro^ also shows promise towards the development of novel inhibitors [10,11].

Several proteins and complexes from SARS-CoV-2 still elude structure determination. The structure of the major structural protein M has yet to be determined, leaving many questions as to how it interacts with both S and its role in viral genome packaging. While the N-terminal domain of Nsp2 has been determined, a structure of the full-length protein structure has yet to be published. Additional non-structural proteins that have not had their structures determined include Nsp4, Nsp6, and Nsp11. Another complex of interest that eludes structure determination is the membrane pore complex in DMVs. Amongst the accessory proteins, ORF3b, ORF7, ORF7b, ORF9c, and ORF10 have yet to be described structurally. In addition, while structures of ORF7a and ORF8 have been determined, structural insight into their mechanisms of action within the host remain unknown [103,104]. As structural techniques improve, it will become more feasible to address the structures of these elusive proteins and their interactions with host factors. Further work on the molecular architecture of SARS-CoV-2 proteins and their host factor interactions could provide the foundation for new developments in antiviral therapies and vaccines, such as work to further stabilize S in its prefusion conformation [109,114].

## Conflict of interest statement

Nothing declared.

## References and recommended reading

Papers of particular interest, published within the period of review, have been highlighted as:

• of special interest
•• of outstanding interest

## References

[1] Feng D., De Vlas S.J., Fang L.Q., Han X.N., Zhao W.J., Sheng S., Yang H., Jia Z.W., Richardus J.H., Cao W.C. (2009). The SARS epidemic in mainland China: bringing together all epidemiological data. Trop Med Int Health.

[2] Drosten C., Gunther S., Preiser W., van der Werf S., Brodt H.R., Becker S., Rabenau H., Panning M., Kolesnikova L., Fouchier R.A. (2003). Identification of a novel coronavirus in patients with severe acute respiratory syndrome. N Engl J Med.

[3] Ksiazek T.G., Erdman D., Goldsmith C.S., Zaki S.R., Peret T., Emery S., Tong S., Urbani C., Comer J.A., Lim W. (2003). A novel coronavirus associated with severe acute respiratory syndrome. N Engl J Med.

[4] Peiris J.S.M., Lai S.T., Poon L.L.M., Guan Y., Yam L.Y.C., Lim W., Nicholls J., Yee W.K.S., Yan W.W., Cheung M.T. (2003). Coronavirus as a possible cause of severe acute respiratory syndrome. Lancet.

[5] Petersen E., Koopmans M., Go U., Hamer D.H., Petrosillo N., Castelli F., Storgaard M., Al Khalili S., Simonsen L. (2020). Comparing SARS-CoV-2 with SARS-CoV and influenza pandemics. Lancet Infect Dis.

[6] Krammer F. (2020). SARS-CoV-2 vaccines in development. Nature.

[7••] Wrapp D., Wang N., Corbett K.S., Goldsmith J.A., Hsieh C.-L., Abiona O., Graham B.S., McLellan J.S. (2020). Cryo-EM structure of the 2019-nCoV spike in the prefusion conformation. Science.

[8•] Zhang L., Lin D., Sun X., Curth U., Drosten C., Sauerhering L., Becker S., Rox K., Hilgenfeld R. (2020). Crystal structure of SARS-CoV-2 main protease provides a basis for design of improved a-ketoamide inhibitors. Science.

[9] Bangaru S., Ozorowski G., Turner H.L., Antanasijevic A., Huang D., Wang X., Torres J.L., Diedrich J.K., Tian J.H., Portnoff A.D. (2020). Structural analysis of full-length SARS-CoV-2 spike protein from an advanced vaccine candidate. Science.

[10] Klemm T., Ebert G., Calleja D.J., Allison C.C., Richardson L.W., Bernardini J.P., Lu B.G., Kuchel N.W., Grohmann C., Shibata Y. (2020). Mechanism and inhibition of the papain-like protease, PLpro, of SARS-CoV-2. EMBO J.

[11] Osipiuk J., Azizi S.A., Dvorkin S., Endres M., Jedrzejczak R., Jones K.A., Kang S., Kathayat R.S., Kim Y., Lisnyak V.G. (2021). Structure of papain-like protease from SARS-CoV-2 and its complexes with non-covalent inhibitors. Nat Commun.

[12] Peng Q., Peng R., Yuan B., Wang M., Zhao J., Fu L., Qi J., Shi Y. (2021). Structural basis of SARS-CoV-2 polymerase inhibition by favipiravir. Innovation (United States).

[13••] Wang Q., Wu J., Wang H., Gao Y., Liu Q., Mu A., Ji W., Yan L., Zhu Y., Zhu C. (2020). Structural basis for RNA replication by the SARS-CoV-2 polymerase. Cell.

[14] Corbett K.S., Edwards D.K., Leist S.R., Abiona O.M., Boyoglu-Barnum S., Gillespie R.A., Himansu S., Schäfer A., Ziwawo C.T., DiPiazza A.T. (2020). SARS-CoV-2 mRNA vaccine design enabled by prototype pathogen preparedness. Nature.

[15] Polack F.P., Thomas S.J., Kitchin N., Absalon J., Gurtman A., Lockhart S., Perez J.L., Pérez Marc G., Moreira E.D., Zerbini C. (2020). Safety and efficacy of the BNT162b2 mRNA Covid-19 vaccine. N Engl J Med.

[16••] Jin Z., Du X., Xu Y., Deng Y., Liu M., Zhao Y., Zhang B., Li X., Zhang L., Peng C. (2020). Structure of Mpro from SARS-CoV-2 and discovery of its inhibitors. Nature.

[17] Zhang P. (2019). Advances in cryo-electron tomography and subtomogram averaging and classification. Curr Opin Struct Biol.

[18] Schur F.K.M., Obr M., Hagen W.J.H., Wan W., Jakobi A.J., Kirkpatrick J.M., Sachse C., Kräusslich H.-G., Briggs J.A.G. (2016). An atomic model of HIV-1 capsid-SP1 reveals structures regulating assembly and maturation. Science.

[19] Mendonça L., Sun D., Ning J., Liu J., Kotecha A., Olek M., Frosio T., Fu X., Himes B.A., Kleinpeter A.B. (2021). CryoET structures of immature HIV gag reveal six-helix bundle. Commun Biol.

[20] Himes B.A., Zhang P. (2018). emClarity: software for high-resolution cryo-electron tomography and subtomogram averaging. Nat Methods.

[21] Tegunov D., Xue L., Dienemann C., Cramer P., Mahamid J. (2021). Multi-particle cryo-EM refinement with M visualizes ribosome-antibiotic complex at 3.5Å in cells. Nat Methods.

[22] Sutton G., Sun D., Fu X., Kotecha A., Hecksel C.W., Clare D.K., Zhang P., Stuart D.I., Boyce M. (2020). Assembly intermediates of orthoreovirus captured in the cell. Nat Commun.

[23••] Ke Z., Oton J., Qu K., Cortese M., Zila V., McKeane L., Nakane T., Zivanov J., Neufeldt C.J., Cerikan B. (2020). Structures and distributions of SARS-CoV-2 spike proteins on intact virions. Nature.

[24••] Yao H., Song Y., Chen Y., Wu N., Xu J., Sun C., Zhang J., Weng T., Zhang Z., Wu Z. (2020). Molecular architecture of the SARS-CoV-2 virus. Cell.

[25•] Liu C., Mendonça L., Yang Y., Gao Y., Shen C., Liu J., Ni T., Ju B., Liu C., Tang X. (2020). The architecture of inactivated SARS-CoV-2 with postfusion spikes revealed by Cryo-EM and Cryo-ET. Structure.

[26•] Turoňová B., Sikora M., Schürmann C., Hagen W.J.H., Welsch S., Blanc F.E.C., von Bülow S., Gecht M., Bagola K., Hörner C. (2020). In situ structural analysis of SARS-CoV-2 spike reveals flexibility mediated by three hinges. Science.

[27] Watanabe Y., Allen J.D., Wrapp D., McLellan J.S., Crispin M. (2020). Site-specific glycan analysis of the SARS-CoV-2 spike. Science.

[28] Du S., Cao Y., Zhu Q., Yu P., Qi F., Wang G., Du X., Bao L., Deng W., Zhu H. (2020). Structurally resolved SARS-CoV-2 antibody shows high efficacy in severely infected hamsters and provides a potent cocktail pairing strategy. Cell.

[29] Zost S.J., Gilchuk P., Case J.B., Binshtein E., Chen R.E., Nkolola J.P., Schäfer A., Reidy J.X., Trivette A., Nargi R.S. (2020). Potently neutralizing and protective human antibodies against SARS-CoV-2. Nature.

[30] Robbiani D.F., Gaebler C., Muecksch F., Lorenzi J.C.C., Wang Z., Cho A., Agudelo M., Barnes C.O., Gazumyan A., Finkin S. (2020). Convergent antibody responses to SARS-CoV-2 in convalescent individuals. Nature.

[31] Letko M., Marzi A., Munster V. (2020). Functional assessment of cell entry and receptor usage for SARS-CoV-2 and other lineage B betacoronaviruses. Nat Microbiol.

[32••] Cai Y., Zhang J., Xiao T., Peng H., Sterling S.M., Walsh R.M., Rawson S., Rits-Volloch S., Chen B. (2020). Distinct conformational states of SARS-CoV-2 spike protein. Science.

[33] Zhu X., Mannar D., Srivastava S.S., Berezuk A.M., Demers J.P., Saville J.W., Leopold K., Li W., Dimitrov D.S., Tuttle K.S. (2021). Cryo-electron microscopy structures of the N501Y SARS-CoV-2 spike protein in complex with ACE2 and 2 potent neutralizing antibodies. PLoS Biol.

[34•] Xu C., Wang Y., Liu C., Zhang C., Han W., Hong X., Wang Y., Hong Q., Wang S., Zhao Q. (2021). Conformational dynamics of SARS-CoV-2 trimeric spike glycoprotein in complex with receptor ACE2 revealed by cryo-EM. Sci Adv.

[35] Yan R., Zhang Y., Li Y., Xia L., Guo Y., Zhou Q. (2020). Structural basis for the recognition of SARS-CoV-2 by full-length human ACE2. Science.

[36] Lan J., Ge J., Yu J., Shan S., Zhou H., Fan S., Zhang Q., Shi X., Wang Q., Zhang L. (2020). Structure of the SARS-CoV-2 spike receptor-binding domain bound to the ACE2 receptor. Nature.

[37] Shang J., Ye G., Shi K., Wan Y., Luo C., Aihara H., Geng Q., Auerbach A., Li F. (2020). Structural basis of receptor recognition by SARS-CoV-2. Nature.

[38] Lv Z., Deng Y.Q., Ye Q., Cao L., Sun C.Y., Fan C., Huang W., Sun S., Sun Y., Zhu L. (2020). Structural basis for neutralization of SARS-CoV-2 and SARS-CoV by a potent therapeutic antibody. Science.

[39•] Zhou D., Duyvesteyn H.M.E., Chen C.P., Huang C.G., Chen T.H., Shih S.R., Lin Y.C., Cheng C.Y., Cheng S.H., Huang Y.C. (2020). Structural basis for the neutralization of SARS-CoV-2 by an antibody from a convalescent patient. Nat Struct Mol Biol.

[40•] Dejnirattisai W., Zhou D., Ginn H.M., Duyvesteyn H.M.E., Supasa P., Case J.B., Zhao Y., Walter T.S., Mentzer A.J., Liu C. (2021). The antigenic anatomy of SARS-CoV-2 receptor binding domain. Cell.

[41] Huo J., Le Bas A., Ruza R.R., Duyvesteyn H.M.E., Mikolajek H., Malinauskas T., Tan T.K., Rijal P., Dumoux M., Ward P.N. (2020). Neutralizing nanobodies bind SARS-CoV-2 spike RBD and block interaction with ACE2. Nat Struct Mol Biol.

[42] Huo J., Zhao Y., Ren J., Zhou D., Duyvesteyn H.M.E., Ginn H.M., Carrique L., Malinauskas T., Ruza R.R., Shah P.N.M. (2020). Neutralization of SARS-CoV-2 by destruction of the prefusion spike. Cell Host Microbe.

[43] Starr T.N., Czudnochowski N., Liu Z., Zatta F., Park Y.J., Addetia A., Pinto D., Beltramello M., Hernandez P., Greaney A.J. (2021). SARS-CoV-2 RBD antibodies that maximize breadth and resistance to escape. Nature.

[44] Barnes C.O., Jette C.A., Abernathy M.E., Dam K.A., Esswein S.R., Gristick H.B., Malyutin A.G., Sharaf N.G., Huey-Tubman K.E., Lee Y.E. (2020). SARS-CoV-2 neutralizing antibody structures inform therapeutic strategies. Nature.

[45] Sun D., Sang Z., Kim Y.J., Xiang Y., Cohen T., Belford A.K., Huet A., Conway J.F., Sun J., Taylor D.J. (2021). Potent neutralizing nanobodies resist convergent circulating variants of SARS-CoV-2 by targeting diverse and conserved epitopes. Nat Commun.

[46] Yang Z., Wang Y., Jin Y., Zhu Y., Wu Y., Li C., Kong Y., Song W., Tian X., Zhan W. (2021). A non-ACE2 competing human single-domain antibody confers broad neutralization against SARS-CoV-2 and circulating variants. Signal Transduct Target Ther.

[47] Banach B.B., Cerutti G., Fahad A.S., Shen C.H., Oliveira De Souza M., Katsamba P.S., Tsybovsky Y., Wang P., Nair M.S., Huang Y. (2021). Paired heavy- and light-chain signatures contribute to potent SARS-CoV-2 neutralization in public antibody responses. Cell Rep.

[48] Ahmad J., Jiang J., Boyd L.F., Zeher A., Huang R., Xia D., Natarajan K., Margulies D.H. (2021). Structures of synthetic nanobody-SARS-CoV-2 receptor-binding domain complexes reveal distinct sites of interaction. J Biol Chem.

[49] Errico J.M., Zhao H., Chen R.E., Liu Z., Case J.B., Ma M., Schmitz A.J., Rau M.J., Fitzpatrick J.A.J., Shi P.Y. (2021). Structural mechanism of SARS-CoV-2 neutralization by two murine antibodies targeting the RBD. Cell Rep.

[50] Kramer K.J., Johnson N.V., Shiakolas A.R., Suryadevara N., Periasamy S., Raju N., Williams J.K., Wrapp D., Zost S.J., Walker L.M. (2021). Potent neutralization of SARS-CoV-2 variants of concern by an antibody with an uncommon genetic signature and structural mode of spike recognition. Cell Rep.

[51] Cerutti G., Rapp M., Guo Y., Bahna F., Bimela J., Reddem E.R., Yu J., Wang P., Liu L., Huang Y. (2021). Structural basis for accommodation of emerging B.1.351 and B.1.1.7 variants by two potent SARS-CoV-2 neutralizing antibodies. Structure.

[52] Yan R., Wang R., Ju B., Yu J., Zhang Y., Liu N., Wang J., Zhang Q., Chen P., Zhou B. (2021). Structural basis for bivalent binding and inhibition of SARS-CoV-2 infection by human potent neutralizing antibodies. Cell Res.

[53] Song D., Wang W., Dong C., Ning Z., Liu X., Liu C., Du G., Sha C., Wang K., Lu J. (2021). Structure and function analysis of a potent human neutralizing antibody CA521FALA against SARS-CoV-2. Commun Biol.

[54] Fedry J., Hurdiss D.L., Wang C., Li W., Obal G., Drulyte I., Du W., Howes S.C., van Kuppeveld F.J.M., Förster F. (2021). Structural insights into the cross-neutralization of SARS-CoV and SARS-CoV-2 by the human monoclonal antibody 47D. Sci Adv.

[55] Chi X., Yan R., Zhang J., Zhang G., Zhang Y., Hao M., Zhang Z., Fan P., Dong Y., Yang Y. (2020). A neutralizing human antibody binds to the N-terminal domain of the spike protein of SARS-CoV-2. Science.

[56] Zhang L., Cao L., Gao X.S., Zheng B.Y., Deng Y.Q., Li J.X., Feng R., Bian Q., Guo X.L., Wang N. (2021). A proof of concept for neutralizing antibody-guided vaccine design against SARS-CoV-2. Natl Sci Rev.

[57] Cerutti G., Guo Y., Wang P., Nair M.S., Wang M., Huang Y., Yu J., Liu L., Katsamba P.S., Bahna F. (2021). Neutralizing antibody 5-7 defines a distinct site of vulnerability in SARS-CoV-2 spike N-terminal domain. Cell Rep.

[58] Cerutti G., Guo Y., Zhou T., Gorman J., Lee M., Rapp M., Reddem E.R., Yu J., Bahna F., Bimela J. (2021). Potent SARS-CoV-2 neutralizing antibodies directed against spike N-terminal domain target a single supersite. Cell Host Microbe.

[59] Pinto D., Park Y.J., Beltramello M., Walls A.C., Tortorici M.A., Bianchi S., Jaconi S., Culap K., Zatta F., De Marco A. (2020). Cross-neutralization of SARS-CoV-2 by a human monoclonal SARS-CoV antibody. Nature.

[60] Rapp M., Guo Y., Reddem E.R., Yu J., Liu L., Wang P., Cerutti G., Katsamba P., Bimela J.S., Bahna F.A. (2021). Modular basis for potent SARS-CoV-2 neutralization by a prevalent VH1-2-derived antibody class. Cell Rep.

[61] Du S., Liu P., Zhang Z., Xiao T., Yasimayi A., Huang W., Wang Y., Cao Y., Xie X.S., Xiao J. (2021). Structures of SARS-CoV-2 B.1.351 neutralizing antibodies provide insights into cocktail design against concerning variants. Cell Res.

[62] Walls A.C., Park Y.J., Tortorici M.A., Wall A., McGuire A.T., Veesler D. (2020). Structure, function, and antigenicity of the SARS-CoV-2 spike glycoprotein. Cell.

[63] Fan X., Cao D., Kong L., Zhang X. (2020). Cryo-EM analysis of the post-fusion structure of the SARS-CoV spike glycoprotein. Nat Commun.

[64•] Mandala V.S., McKay M.J., Shcherbakov A.A., Dregni A.J., Kolocouris A., Hong M. (2020). Structure and drug binding of the SARS-CoV-2 envelope protein transmembrane domain in lipid bilayers. Nat Struct Mol Biol.

[65] Nieto-Torres J.L., DeDiego M.L., Verdiá-Báguena C., Jimenez-Guardeño J.M., Regla-Nava J.A., Fernandez-Delgado R., Castaño-Rodriguez C., Alcaraz A., Torres J., Aguilella V.M. (2014). Severe acute respiratory syndrome coronavirus envelope protein ion channel activity promotes virus fitness and pathogenesis. PLoS Pathog.

[66] Mu J., Xu J., Zhang L., Shu T., Wu D., Huang M., Ren Y., Li X., Geng Q., Xu Y. (2020). SARS-CoV-2-encoded nucleocapsid protein acts as a viral suppressor of RNA interference in cells. Sci China Life Sci.

[67] Peng Y., Du N., Lei Y., Dorje S., Qi J., Luo T., Gao G.F., Song H. (2020). Structures of the SARS-CoV-2 nucleocapsid and their perspectives for drug design. EMBO J.

[68] Chang C.-K., Hsu Y.-L., Chang Y.-H., Chao F.-A., Wu M.-C., Huang Y.-S., Hu C.-K., Huang T.-H. (2009). Multiple nucleic acid binding sites and intrinsic disorder of severe acute respiratory syndrome coronavirus nucleocapsid protein: implications for ribonucleocapsid protein packaging. J Virol.

[69] Ye Q., West A.M.V., Silletti S., Corbett K.D. (2020). Architecture and self-assembly of the SARS-CoV-2 nucleocapsid protein. Protein Sci.

[70] Kang S., Yang M., Hong Z., Zhang L., Huang Z., Chen X., He S., Zhou Z., Zhou Z., Chen Q. (2020). Crystal structure of SARS-CoV-2 nucleocapsid protein RNA binding domain reveals potential unique drug targeting sites. Acta Pharm Sin B.

[71] Zinzula L., Basquin J., Bohn S., Beck F., Klumpe S., Pfeifer G., Nagy I., Bracher A., Hartl F.U., Baumeister W. (2021). High-resolution structure and biophysical characterization of the nucleocapsid phosphoprotein dimerization domain from the Covid-19 severe acute respiratory syndrome coronavirus 2. Biochem Biophys Res Commun.

[72] Klein S., Cortese M., Winter S.L., Wachsmuth-Melm M., Neufeldt C.J., Cerikan B., Stanifer M.L., Boulant S., Bartenschlager R., Chlanda P. (2020). SARS-CoV-2 structure and replication characterized by in situ cryo-electron tomography. Nat Commun.

[73] Ullrich S., Nitsche C. (2020). The SARS-CoV-2 main protease as drug target. Bioorg Med Chem Lett.

[74] Lee J., Worrall L.J., Vuckovic M., Rosell F.I., Gentile F., Ton A.T., Caveney N.A., Ban F., Cherkasov A., Paetzel M. (2020). Crystallographic structure of wild-type SARS-CoV-2 main protease acyl-enzyme intermediate with physiological C-terminal autoprocessing site. Nat Commun.

[75] Douangamath A., Fearon D., Gehrtz P., Krojer T., Lukacik P., Owen C.D., Resnick E., Strain-Damerell C., Aimon A., Abranyi-Balogh P. (2020). Crystallographic and electrophilic fragment screening of the SARS-CoV-2 main protease. Nat Commun.

[76] Shin D., Mukherjee R., Grewe D., Bojkova D., Baek K., Bhattacharya A., Schulz L., Widera M., Mehdipour A.R., Tascher G. (2020). Papain-like protease regulates SARS-CoV-2 viral spread and innate immunity. Nature.

[77] Schubert K., Karousis E.D., Jomaa A., Scaiola A., Echeverria B., Gurzeler L.A., Leibundgut M., Thiel V., Mühlemann O., Ban N. (2020). SARS-CoV-2 Nsp1 binds the ribosomal mRNA channel to inhibit translation. Nat Struct Mol Biol.

[78] Thoms M., Buschauer R., Ameismeier M., Koepke L., Denk T., Hirschenberger M., Kratzat H., Hayn M., Mackens-Kiani T., Cheng J. (2020). Structural basis for translational shutdown and immune evasion by the Nsp1 protein of SARS-CoV-2. Science.

[79] Yuan S., Peng L., Park J.J., Hu Y., Devarkar S.C., Dong M.B., Shen Q., Wu S., Chen S., Lomakin I.B. (2020). Nonstructural protein 1 of SARS-CoV-2 is a potent pathogenicity factor redirecting host protein synthesis machinery toward viral RNA. Mol Cell.

[80] Ma J., Chen Y., Wu W., Chen Z. (2021). Structure and function of N-terminal zinc finger domain of SARS-CoV-2 NSP2. Virol Sin.

[81] Lin M.H., Chang S.C., Chiu Y.C., Jiang B.C., Wu T.H., Hsu C.H. (2020). Structural, biophysical, and biochemical elucidation of the SARS-CoV-2 nonstructural protein 3 macro domain. ACS Infect Dis.

[82] Michalska K., Kim Y., Jedrzejczak R., Maltseva N.I., Stols L., Endres M., Joachimiak A. (2020). Crystal structures of SARS-CoV-2 ADP-ribose phosphatase: from the apo form to ligand complexes. IUCrJ.

[83] Frick D.N., Virdi R.S., Vuksanovic N., Dahal N., Silvaggi N.R. (2020). Molecular basis for ADP-ribose binding to the Mac1 domain of SARS-CoV-2 nsp3. Biochemistry.

[84] Ni X., Schröder M., Olieric V., Sharpe M.E., Hernandez-Olmos V., Proschak E., Merk D., Knapp S., Chaikuad A. (2021). Structural insights into plasticity and discovery of remdesivir metabolite GS-441524 binding in SARS-CoV-2 macrodomain. ACS Med Chem Lett.

[85] Gao Y., Yan L., Huang Y., Liu F., Zhao Y., Cao L., Wang T., Sun Q., Ming Z., Zhang L. (2020). Structure of the RNA-dependent RNA polymerase from COVID-19 virus. Science.

[86] Peng Q., Peng R., Yuan B., Zhao J., Wang M., Wang X., Wang Q., Sun Y., Fan Z., Qi J. (2020). Structural and biochemical characterization of the nsp12-nsp7-nsp8 core polymerase complex from SARS-CoV-2. Cell Rep.

[87] Hillen H.S., Kokic G., Farnung L., Dienemann C., Tegunov D., Cramer P. (2020). Structure of replicating SARS-CoV-2 polymerase. Nature.

[88] Chen J., Malone B., Llewellyn E., Grasso M., Shelton P.M.M., Olinares P.D.B., Maruthi K., Eng E.T., Vatandaslar H., Chait B.T. (2020). Structural basis for helicase-polymerase coupling in the SARS-CoV-2 replication-transcription complex. Cell.

[89] Yan L., Zhang Y., Ge J., Zheng L., Gao Y., Wang T., Jia Z., Wang H., Huang Y., Li M. (2020). Architecture of a SARS-CoV-2 mini replication and transcription complex. Nat Commun.

[90] Yan L., Ge J., Zheng L., Zhang Y., Gao Y., Wang T., Huang Y., Yang Y., Gao S., Li M. (2021). Cryo-EM structure of an extended SARS-CoV-2 replication and transcription complex reveals an intermediate state in cap synthesis. Cell.

[91] Malone B., Chen J., Wang Q., Llewellyn E., Choi Y.J., Olinares P.D.B., Cao X., Hernandez C., Eng E.T., Chait B.T. (2021). Structural basis for backtracking by the SARS-CoV-2 replication-transcription complex. Proc Natl Acad Sci U S A.

[92] Littler D.R., Gully B.S., Colson R.N., Rossjohn J. (2020). Crystal structure of the SARS-CoV-2 non-structural protein 9, Nsp9. iScience.

[93] Rogstam A., Nyblom M., Christensen S., Sele C., Talibov V.O., Lindvall T., Rasmussen A.A., André I., Fisher Z., Knecht W. (2020). Crystal structure of non-structural protein 10 from severe acute respiratory syndrome coronavirus-2. Int J Mol Sci.

[94] Lin S., Chen H., Chen Z., Yang F., Ye F., Zheng Y., Yang J., Lin X., Sun H., Wang L. (2021). Crystal structure of SARS-CoV-2 nsp10 bound to nsp14-ExoN domain reveals an exoribonuclease with both structural and functional integrity. Nucleic Acids Res.

[95••] Yan L., Yang Y., Li M., Zhang Y., Zheng L., Ge J., Huang Y.C., Liu Z., Wang T., Gao S. (2021). Coupling of N7-methyltransferase and 3′-5′ exoribonuclease with SARS-CoV-2 polymerase reveals mechanisms for capping and proofreading. Cell.

[96] Lin S., Chen H., Ye F., Chen Z., Yang F., Zheng Y., Cao Y., Qiao J., Yang S., Lu G. (2020). Crystal structure of SARS-CoV-2 nsp10/nsp16 2′-O-methylase and its implication on antiviral drug design. Signal Transduct Target Ther.

[97] Rosas-Lemus M., Minasov G., Shuvalova L., Inniss N.L., Kiryukhina O., Brunzelle J., Satchell K.J.F. (2020). High-resolution structures of the SARS-CoV-2 2’-*O*-methyltransferase reveal strategies for structure-based inhibitor design. Sci Signal.

[98] Viswanathan T., Arya S., Chan S.H., Qi S., Dai N., Misra A., Park J.G., Oladunni F., Kovalskyy D., Hromas R.A. (2020). Structural basis of RNA cap modification by SARS-CoV-2. Nat Commun.

[99] Kim Y., Jedrzejczak R., Maltseva N.I., Wilamowski M., Endres M., Godzik A., Michalska K., Joachimiak A. (2020). Crystal structure of Nsp15 endoribonuclease NendoU from SARS-CoV-2. Protein Sci.

[100] Pillon M.C., Frazier M.N., Dillard L.B., Williams J.G., Kocaman S., Krahn J.M., Perera L., Hayne C.K., Gordon J., Stewart Z.D. (2021). Cryo-EM structures of the SARS-CoV-2 endoribonuclease Nsp15 reveal insight into nuclease specificity and dynamics. Nat Commun.

[101] Kim Y., Wower J., Maltseva N., Chang C., Jedrzejczak R., Wilamowski M., Kang S., Nicolaescu V., Randall G., Michalska K. (2021). Tipiracil binds to uridine site and inhibits Nsp15 endoribonuclease NendoU from SARS-CoV-2. Commun Biol.

[102] Kern D.M., Sorum B., Mali S.S., Hoel C.M., Sridharan S., Remis J.P., Toso D.B., Kotecha A., Bautista D.M., Brohawn S.G. (2021). Cryo-EM structure of SARS-CoV-2 ORF3a in lipid nanodiscs. Nat Struct Mol Biol.

[103] Zhou Z., Huang C., Zhou Z., Huang Z., Su L., Kang S., Chen X., Chen Q., He S., Rong X. (2021). Structural insight reveals SARS-CoV-2 ORF7a as an immunomodulating factor for human CD14(+) monocytes. iScience.

[104] Flower T.G., Buffalo C.Z., Hooy R.M., Allaire M., Ren X., Hurley J.H. (2021). Structure of SARS-CoV-2 ORF8, a rapidly evolving immune evasion protein. Proc Natl Acad Sci U S A.

[105] Gao X., Zhu K., Qin B., Olieric V., Wang M., Cui S. (2021). Crystal structure of SARS-CoV-2 Orf9b in complex with human TOM70 suggests unusual virus-host interactions. Nat Commun.

[106] Gordon D.E., Hiatt J., Bouhaddou M., Rezelj V.V., Ulferts S., Braberg H., Jureka A.S., Obernier K., Guo J.Z., Batra J. (2020). Comparative host-coronavirus protein interaction networks reveal pan-viral disease mechanisms. Science.

[107••] Mendonça L., Howe A., Gilchrist J.B., Sheng Y., Sun D., Knight M.L., Zanetti-Domingues L.C., Bateman B., Krebs A.-S., Chen L. (2021). Correlative multi-scale cryo-imaging unveils SARS-CoV-2 assembly and egress. Nat Commun.

[108•] Wolff G., Limpens R.W.A.L., Zevenhoven-Dobbe J.C., Laugks U., Zheng S., de Jong A.W.M., Koning R.I., Agard D.A., Grünewald K., Koster A.J. (2020). A molecular pore spans the double membrane of the coronavirus replication organelle. Science.

[109] Pallesen J., Wang N., Corbett K.S., Wrapp D., Kirchdoerfer R.N., Turner H.L., Cottrell C.A., Becker M.M., Wang L., Shi W. (2017). Immunogenicity and structures of a rationally designed prefusion MERS-CoV spike antigen. Proce Natl Acad Sci U S A.

[110] Watanabe Y., Mendonça L., Allen E.R., Howe A., Lee M., Allen J.D., Chawla H., Pulido D., Donnellan F., Davies H. (2021). Native-like SARS-CoV-2 spike glycoprotein expressed by ChAdOx1 nCoV-19/AZD1222 vaccine. ACS Cent Sci.

[111] Vandyck K., Deval J. (2021). Considerations for the discovery and development of 3-chymotrypsin-like cysteine protease inhibitors targeting SARS-CoV-2 infection. Curr Opin Virol.

[112] Cai Y., Zhang J., Xiao T., Lavine C.L., Rawson S., Peng H., Zhu H., Anand K., Tong P., Gautam A. (2021). Structural basis for enhanced infectivity and immune evasion of SARS-CoV-2 variants. Science.

[113] Kabinger F., Stiller C., Schmitzova J., Dienemann C., Kokic G., Hillen H.S., Hobartner C., Cramer P. (2021). Mechanism of molnupiravir-induced SARS-CoV-2 mutagenesis. Nat Struct Mol Biol.

[114] Hsieh C.-L., Goldsmith J.A., Schaub J.M., DiVenere A.M., Kuo H.-C., Javanmardi K., Le K.C., Wrapp D., Lee A.G., Liu Y. (2020). Structure-based design of prefusion-stabilized SARS-CoV-2 spikes. Science.

